# Climatically driven fluctuations in Southern Ocean ecosystems

**DOI:** 10.1098/rspb.2007.1180

**Published:** 2007-10-17

**Authors:** Eugene J Murphy, Philip N Trathan, Jon L Watkins, Keith Reid, Michael P Meredith, Jaume Forcada, Sally E Thorpe, Nadine M Johnston, Peter Rothery

**Affiliations:** 1 British Antarctic Survey, Natural Environment Research CouncilHigh Cross, Madingley Road, Cambridge, Cambridgeshire CB3 0ET, UK; 2 Centre for Ecology and Hydrology, Natural Environment Research CouncilMonks Wood, Abbots Ripton, Huntingdon, Cambridgeshire PE28 2LS, UK

**Keywords:** Southern Ocean, ecosystem, krill, predators, climate, El Niño Southern Oscillation

## Abstract

Determining how climate fluctuations affect ocean ecosystems requires an understanding of how biological and physical processes interact across a wide range of scales. Here we examine the role of physical and biological processes in generating fluctuations in the ecosystem around South Georgia in the South Atlantic sector of the Southern Ocean. Anomalies in sea surface temperature (SST) in the South Pacific sector of the Southern Ocean have previously been shown to be generated through atmospheric teleconnections with El Niño Southern Oscillation (ENSO)-related processes. These SST anomalies are propagated via the Antarctic Circumpolar Current into the South Atlantic (on time scales of more than 1 year), where ENSO and Southern Annular Mode-related atmospheric processes have a direct influence on short (less than six months) time scales. We find that across the South Atlantic sector, these changes in SST, and related fluctuations in winter sea ice extent, affect the recruitment and dispersal of Antarctic krill. This oceanographically driven variation in krill population dynamics and abundance in turn affects the breeding success of seabird and marine mammal predators that depend on krill as food. Such propagating anomalies, mediated through physical and trophic interactions, are likely to be an important component of variation in ocean ecosystems and affect responses to longer term change. Population models derived on the basis of these oceanic fluctuations indicate that plausible rates of regional warming of 1^o^C over the next 100 years could lead to more than a 95% reduction in the biomass and abundance of krill across the Scotia Sea by the end of the century.

## 1. Introduction

Climate processes are a major determinant of the structure and function of ecological systems (McGowan *et al*. 1998; Stenseth *et al*. 2002). A wide range of studies have shown links between fluctuations in climate and ecological processes in terrestrial, freshwater and marine ecosystems (McGowan *et al*. 1998; Attrill & Power 2002; Stenseth *et al*. 2002; Quetin & Ross 2003). In marine ecosystems, the impact of climate-related physical fluctuations can be both direct: through local atmospheric effects, and indirect: through remote changes in oceanic or sea ice processes that show marked coherent spatial and temporal variability (Stenseth *et al*. 2003; Pershing *et al*. 2004). As a result, interannual and sub-decadal fluctuations in large-scale climate processes, such as the El Niño Southern Oscillation (ENSO), the North Atlantic Oscillation (NAO) and the Pacific Decadal Oscillation, can have a strong influence on local ecological processes in oceanic ecosystems (Stenseth *et al*. 2002, 2003). Understanding how ecosystems respond to such climate-driven oceanic variation is required for predicting long-term responses to change and management of fisheries (Attrill & Power 2002; Ottersen *et al*. 2004*a*,*b*).

The impacts of climate-related fluctuations are difficult to distinguish owing to the complexity of marine ecosystems (Stenseth *et al*. 2002, 2003). The multiple scales of interaction between physical and biological processes, and within biological systems, generate complex interaction and feedback effects (Stenseth *et al*. 2002). Biologically, individuals and populations can be affected directly through physiology or indirectly via the food web through prey, predator and competitive interactions. These processes can generate a range of impacts on populations and communities, including variable effects on males or females or on different stages of life cycles, delays through variation in cohort success or feedbacks in food webs, density-dependent population effects and changes in the frequency of population fluctuation or species interaction (Royama 1992; Ottersen & Stenseth 2001; Stenseth *et al*. 2002, 2003; Ottersen *et al*. 2004*a*,*b*).

One region in which the effect of climate fluctuations on ocean ecosystems has been extensively studied is the North Atlantic. Recent reviews have considered the complex indirect, direct and interactive effects of climate on this system (Ottersen *et al*. 2001, 2004*b*). The copepod *Calanus finmarchicus* is a key species in the North Atlantic ecosystem, and an important factor determining its abundance in the shelf areas of the eastern North Atlantic (North Sea) is the extent of advection of this species into the region via the inflow of cooler waters (Pershing *et al*. 2004). This inflow is influenced by the state of the NAO, with cooler waters being dominant when the NAO index is negative. The authors call variations in the inflow of *C. finmarchicus* a direct translation effect that is due to climate-related shifts in advection. Such improved insight into the mechanisms that generate fluctuations in natural systems is key to predicting future change (Stenseth *et al*. 2002).

In the South Atlantic sector of the Southern Ocean, correlations have been noted between fluctuations in the production and survival of penguins, seals and other predators and physical variations (atmospheric and oceanic) in the equatorial Pacific (Croxall *et al*. 1988; Forcada *et al*. 2005). However, although the dynamics of the predator populations have been modelled to take account of the remote fluctuations, so far it has only been possible to speculate on the mechanisms involved in generating these correlations. The upper trophic-level predators that occur in such vast numbers in the Southern Ocean give a view of the overall health of this unique ecosystem. One of the key features of this system is the importance of one particular species, Antarctic krill (*Euphausia superba* Dana; Croxall *et al*. 1988). Antarctic krill (termed krill) have a circumpolar distribution but are most abundant in the Scotia Sea and Antarctic Peninsula regions where they are a key species in the food web and a target of a commercial fishery (Atkinson *et al*. 2004). Here we elucidate some of the mechanisms linking climate variations in the equatorial Pacific and this remote dynamic ecosystem some 10 000 km away. We find that fluctuations in the physical characteristics of the Atlantic sector of the Southern Ocean are associated with Southern Hemisphere-scale oceanic and climatic variation. We also find that the signal is mediated through krill via physical-biological interactions affecting their population dynamics and large-scale distribution which in turn impacts the breeding success of krill-dependent penguins and seals. These results provide a preliminary basis for predictions of the response of these ecosystems to fishing and natural and human-induced climate change.

## 2. Methods and data

### (a) Sea surface temperature and sea ice data

To examine physical variability, we used one-degree resolution monthly National Oceanographic and Atmospheric Administration (NOAA) optimum interpolation (OI) sea surface temperature (SST) V2 and sea ice data (Reynolds *et al*. 2002) from the IRL/LDEO Climate Data Library. To consider the large-scale links between the ENSO and the Southern Ocean, we used two NCEP SST-derived ENSO indices. Nino-4 gives an index of SST variation in the western equatorial Pacific and is the SST anomaly averaged over 5^o^S to 5°N and 160°E to 150°W. Nino-3 gives an index of SST variation in the eastern equatorial Pacific and is the SST anomaly averaged over 5°S to 5°N and 150°W to 90°W. The annual and monthly Southern Annular Mode (SAM) index data (Marshall 2003) were obtained from http://www.nerc-bas.ac.uk/icd/gjma/sam.html.

### (b) Krill populations and climate fluctuations

To examine whether fluctuations in the krill population were related to oceanic variations, we used annual acoustically derived estimates of biomass from South Georgia (see Murphy *et al*. 2007 for references), historically derived estimates of abundance (numerical density) from net sampling across the South Atlantic (Atkinson *et al*. 2004) and estimates of the mean length of krill consumed by Antarctic fur seals (*Arctocephalus gazella*, termed fur seals) at South Georgia during March (Reid *et al*. 1999). We used linear multiple regression models (Coulson *et al*. 2000; Aanes *et al*. 2002) to examine the factors influencing krill population growth rates (abundance and biomass) and changes in length. Maximum-likelihood methods were used for model fitting. This approach examines the relative influence on the dynamics of autoregressive (here we used a single 1-year autoregressive term, based on the natural log of either population density or biomass) and lagged environmental variable effects (we included up to 2-year lags of SST, sea ice concentration and sea ice extent). We used only environmental variables (SST and sea ice) from the South Atlantic sector in the analyses of krill dynamics (see electronic supplementary material for details of timing of variables used). Significant autoregressive terms are sometimes considered to indicate density-dependent effects of changes in mortality or growth in relation to population size. However, these can arise through a range of interaction effects or census issues and may not be the result of population density dependence (Royama 1992; Freckleton *et al*. 2006).

The krill abundance (numerical density) across the region shows a significant negative trend over the last 30 years (Atkinson *et al*. 2004). We therefore removed the exponential decline (ln *N*_*t*_ =153.22−0.0756 *t*, *R*^2^=35%, *p*<0.005) from the abundance series to generate a stationary series (*X*_*t*_), which was then used to derive the *per capita* growth rates (*R*_*t*_), where:

$$R_{t}=X_{t+1}-X_{t}.$$

Akaike Information Criterion (AIC_c_) was applied to consider model fit and identify the simplest most appropriate model in each case (Coulson *et al*. 2000; Aanes *et al*. 2002). Linear models of the form used here can be useful for examining the combined effects of population and climate processes and can give a reasonable representation of nonlinear effects in some populations. However, we also need to understand the mechanisms involved in generating density-dependent and density-independent effects (Coulson *et al*. 2000; Ottersen & Stenseth 2001; Stenseth *et al*. 2002). Here we combine the multiple regression approach with a demographic model analysis to examine some of the mechanisms involved.

Further details of methods used are given in the electronic supplementary material.

## 3. Results and discussion

### (a) Pacific and South Atlantic variations in SST

**Figure 1:**
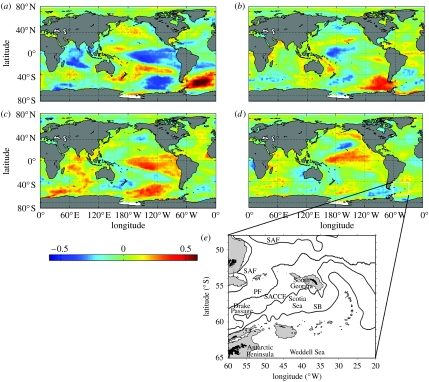
Correlations of anomalies of remotely sensed sea surface temperature (SST) with the SST anomaly variation at South Georgia (35.5°W, 53.5°S). Correlations at (*a*) 0-, (*b*) 12-, (*c*) 24- and (*d*) 36-month lags. The bar shows the correlation (*r*) colour scale. CIs (95%) calculated to take account of autocorrelation (Trenberth 1984) were from 0.33 to 0.35 between the ENSO region and the South Georgia series, 0.28 to 0.3 between the South Georgia series and the South Atlantic and southeast Pacific sectors and approximately 0.25 in the southwest Pacific sector. (*e*) The South Atlantic sector in detail and the approximate position of the major ocean fronts. SAF, Sub-Antarctic Front; PF, Polar Front; SACCF, Southern Antarctic Circumpolar Current Front; SB, Southern Boundary of the Antarctic Circumpolar Current. (*a*–*d*) Correlations of a time series of remotely sensed SST near South Georgia with correlations from all other areas in the world ocean. (*a*) is for zero lag, i.e. the instantaneous correlation. Note the region of high correlation around South Georgia, showing the spatial scale of the coherent oceanic anomalies. Note also the significant correlations with SST in the equatorial Pacific, indicative of the direct connection with the ENSO region. The Pacific–South American teleconnection pattern (alternating positive and negative anomalies extending to high latitudes in the Pacific) is clearly visible. (*b*–*d*) are with progressive yearly lags, and essentially track the anomalies back through time from the region around South Georgia ‘upstream’ into the Pacific. Twelve months before anomalies reach South Georgia, they occupy the southeast Pacific west of Drake Passage, the central South Pacific 24 months earlier and the area in the western South Pacific 36 months earlier.

Signals of ENSO variability in the tropical Pacific are known to propagate to high latitudes through atmospheric teleconnection and oceanic processes (Kwok & Comiso 2002; Liu *et al*. 2002, 2004; White *et al*. 2002; Meredith *et al*. 2004, 2005, in press; Turner 2004). Over recent decades, ENSO-related variation throughout the South Pacific and Atlantic regions has been quasi-cyclic, with the periodicity varying approximately between 4 and 6 years, but with marked variation in anomaly intensity and duration on decadal and longer time scales (White & Peterson 1996; Trathan & Murphy 2002; Carleton 2003; Turner 2004). Southern Ocean atmosphere, ocean and sea ice systems are strongly coupled showing marked variation on time scales ranging from years to decades and between regions (Carleton 2003; Turner 2004). Spatial correlation analysis of the global SST anomaly field with variations at South Georgia in the South Atlantic reveals a wave-like progression (figure 1). As the anomalies propagate across the South Pacific and into the South Atlantic sectors, they are correlated with the changing phases of ENSO in the equatorial region of the Pacific. Changes in atmospheric circulation patterns across the southwest Pacific associated with ENSO variation provide a mechanism for the generation of SST anomalies in the Pacific sector of the Southern Ocean (Li 2000; White *et al*. 2002; Turner 2004). The propagating signal shows consistent correlations (*r*>0.5: 95% significance level for *r* is between approximately 0.25 and 0.33) meridionally across the Antarctic Circumpolar Current (ACC) and over more than 3000 km east–west (figure 1).

Cross-correlation analyses of SST data from locations along 60°S show that positive anomalies in the ENSO region variation occur approximately 2–3 years prior to positive anomalies close to South Georgia (Trathan & Murphy 2002; Meredith *et al*. in press; figure 1 in the electronic supplementary material). The observation of slow propagating anomalies across the South Pacific sector into the Atlantic is consistent with the concept of a propagating anomaly wave (White & Peterson 1996) moving in association with the ACC (Murphy *et al*. 1995; White *et al*. 2002; Venegas 2003). Alternatively, these variations may be the result of spatial differences in the geographical relationships of SST variation across the southern Pacific fluctuating in phase with ENSO (Park *et al*. 2004) or some combination of these processes. The observed pattern of positive anomalies in the southeast Pacific coinciding with negative anomalies in the southwest Atlantic (and vice versa) is also consistent with the operation of the Antarctic Dipole (Yuan & Martinson 2000; Carleton 2003); it may also depend in part on the unusual footprint in the southeast Pacific of the SAM (Meredith *et al*. in press; see Thompson & Solomon 2002 for a discussion of SAM).

The anomalies and their propagation are not simply atmospheric effects impacting locally on the ocean surface. Regional studies show that changes in SST around South Georgia also show variability due to sub-surface changes in temperature and salinity that originate remotely (and over a broad area) several months previously (Meredith *et al*. 2005). Such oceanic advection of temperature anomalies is also evidenced by expendable bathythermograph measurements at Drake Passage (see www.hrx.ucsd.edu). These anomalies propagate across the region into the Atlantic sector generating warm and cold periods generally lasting 2–3 years in particular regions (electronic supplementary material, figures 1 and 2).

**Figure 2:**
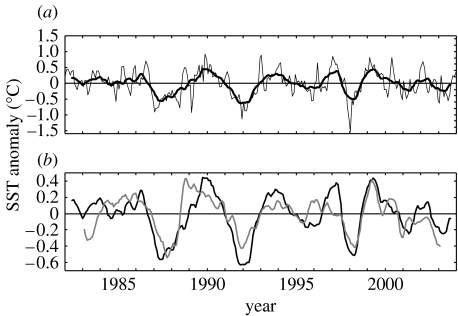
Predicted sea surface temperature (SST) anomaly series at South Georgia derived by multiple regression analysis. (*a*) South Georgia SST anomaly series (thin line) with the 12-month moving average series (bold line). (*b*) Smoothed South Georgia series (black line) with predicted series (grey line) based on the regression relationship with the lagged Bellingshausen Sea series (89.5°W, 60°S), Nino-4 and Nino-3 region series, *R*^2^=30% (*n*=255, *p*<0.001). A four-month moving average was applied to the predicted series to remove high-frequency monthly variability for plotting. (See electronic supplementary material for further details.)

During the intense El Niño period of 1997–1998, atmospheric changes in the ENSO region appeared to have a strong, direct, short-term (less than six months) effect on the Atlantic sector of the Southern Ocean (electronic supplementary material, figures 1 and 2; Turner 2004; Meredith *et al*. 2005). This was confirmed through multiple regression analyses, which showed that a model including both a propagating oceanic signal in the South Pacific sector (10 months prior) and equatorial region variation (four months prior) explained more of the South Atlantic sector variation (*R*^2^=30%; figure 2) than a model just based on the propagating signal of the South Pacific sector (*R*^2^=21%; see also electronic supplementary material). Meredith *et al*. (2004) showed that direct atmospheric forcing associated with the 1997–1998 El Niño dominated the upper ocean stratification at the western Antarctic Peninsula during this period. Multiple regression analyses including the SAM index indicate that the majority of the variations (28% out of 30%) in the Scotia SSTs are explained by a model including both southeast Pacific sector and ENSO region SST variations. Inclusion of the SAM index (Meredith *et al*. in press), or a secondary ENSO index, explains the remaining 2% of the variability (see electronic supplementary material, table 2).

Changes in regional SST are coupled to variations in seasonal sea ice thermodynamics (White & Peterson 1996; Hall & Visbeck 2002; Liu *et al*. 2004; Trathan *et al*. 2006). Within the South Atlantic sector, cold periods around South Georgia (north of the seasonally covered sea ice zone) tend to occur when sea ice is further north in the Scotia Sea during the previous winter and vice versa, such that changes in sea ice distribution lead SST fluctuations by less than six months (electronic supplementary material, figure 3).

### (b) Population dynamics of krill in the Scotia Sea

**Figure 3:**
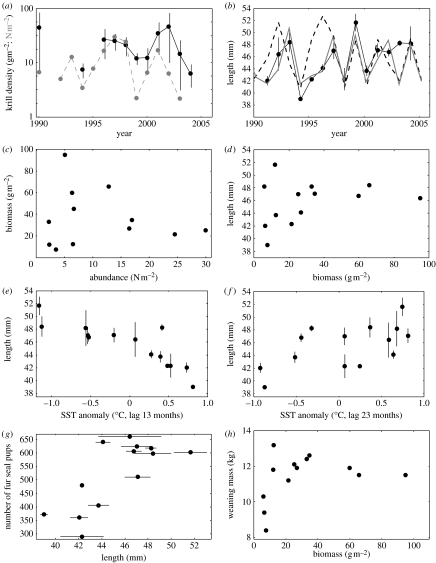
(*a*) Interannual changes in krill biomass (solid black line, in g m^−2^, with 95% CIs) at South Georgia (British Antarctic Survey data) and numerical abundance (Atkinson *et al*. 2004) in the southwest Atlantic sector (grey dashed line, in N m^−2^). (*b*) Interannual changes in mean krill length in the diet of Antarctic fur seals at South Georgia in March (black solid line); CIs (95%) are shown for the mean krill length in March, based on weekly samples (Reid *et al*. 1999). The dashed line shows the model-estimated changes where recruitment of post-larval krill is driven by a functional relationship based on the sea surface temperature (SST) anomaly 2 years previously and the grey line is based on anomalies 1 year previously. (*c*) Relationship between the abundance of krill in the South Atlantic and the biomass of krill at South Georgia. (*d*) Relationship between the mean krill length (in mm) in the diet of Antarctic fur seals at Bird Island, South Georgia (in March) and the biomass of krill at South Georgia. (*e*) Relationship between the mean krill length in the diet of Antarctic fur seals at Bird Island, South Georgia (in March) and SST anomalies in the South Georgia region 13 months earlier (*n*=14, adjusted *n*=12.5; *r*=−0.85, *r*_*s*_=−0.86, *r*_12,0.05_=0.55, *r*_*s*12,0.05_=0.59). (*f*) As given for (*e*) but for 23 months earlier (*n*=14, adjusted *n*=12.9; *r*=0.60, *r*_*s*_=0.60, *r*_12,0.05_=0.55, *r*_*s*12,0.05_=0.59). (*g*) Relationship between the number of Antarctic fur seal pups produced and the mean length of krill (in mm) in the diet of adult Antarctic fur seals at Bird Island, South Georgia. (*h*) Relationship between the Antarctic fur seal pup weaning mass and the biomass of krill at South Georgia.

Having established that interannual variation in Scotia Sea SST and sea ice extent depends significantly upon external forcing, we next examined the impact of these fluctuations on the krill population. We compared krill abundance across the South Atlantic with krill biomass estimates from local surveys at South Georgia (figure 3*a*). Although these data series are short, the pattern of fluctuations shows a delay between a change in numbers and biomass which is consistent with current views of krill population dynamics (Murphy *et al*. 1998; Murphy & Reid 2001). Krill abundance across the South Atlantic sector was lowest when biomass was also low at South Georgia (figure 3*c*). These low biomass periods were also observed when krill length was most variable (figure 3*d*). Animals were more consistent in size (approx. 46–49 mm) during periods of higher biomass.

We examined the influence of physical fluctuations in the South Atlantic on krill population dynamics. We found a strong positive relationship between the population growth rates derived from the biomass series (ln[*B*_*t*+1_*/B*_*t*_], where *B*_*t*_ is the krill biomass in year *t*) and SST 2 years earlier (i.e. 12–24 months after a warm spring, there is an increase in biomass) (table 1, M1). The model identified by AIC_c_ (M2) included only SST variables for the zero-lag spring (negative) and the summer 2 years earlier (positive; M2). There is also a significant secondary influence of population size (*B*_0_), indicating that population effects may also be important (M3 and M4; electronic supplementary material, table 3). However, the magnitude of the regression coefficient for *B*_0_ varied between 0.60 and 1.18, which indicates a highly damped population response to variability and hence very weak direct density dependence. More complex models indicate combined effects of SST and sea ice conditions 2 years earlier but do not include population effects (table 1, M5; electronic supplementary material). There was no indication of a significant delayed density-dependent effect. The dominance of climate-related variations (M1–M5) indicates that the biomass fluctuations are driven mainly by the delayed effects of SST variation 2 years earlier, with an additional influence of physical conditions (SST) in the summer of recruitment (M2). Krill around South Georgia are at least 2 years old, although they are usually considered to be 3 years old when they become the dominant biomass class. This suggests that environmental conditions affecting the production, survival and dispersal of the 0 or 1 age classes have a significant delayed influence on the biomass of krill around South Georgia 2 years later.

Examining the krill population growth rates based on the residual fluctuations in abundance (*X*_*t*+1_−*X*_*t*_), there are significant population (direct density dependence) and environmental effects (SST and sea ice variation; table 1, M6–M10; electronic supplementary material). However, there are also indications that delayed density-dependent effects are a significant influence (M7–M10). This suggests that intrinsic density-dependent population processes are more important in generating fluctuations in abundance than in biomass. The parameter values in the models including both *N*_*t*_ (abundance at time *t*) and *N*_*t*−1_ terms suggest oscillatory dynamics in a declining series. The coefficients for the lag population effects are between −0.5 and −1.0 and the direct effects are approximately −0.75, which correspond to oscillations with a period of approximately 5 years. The application of AIC_c_ identified model M9, which includes terms *N*_*t*_, *N*_*t*−1_ and shows a positive relationship with ice extent 2 years earlier. The coefficients in models M7 and M9 suggest damped population-driven oscillations and indicate that density-dependent effects dominate (*R*^*2*^=61%), while the ice conditions 2 years earlier explain a further 26% of the variation about the trend. More complex three-variable models also indicate that sea ice and SST over the previous 2 years influence population growth rate (table 1; electronic supplementary material, table 3). The influence of SST is sensitive to the inclusion of other model variables, but there are consistent indications that extensive winter sea ice has a positive impact on population abundance 2 years later. This indicates that for the abundance series, the winter conditions during the period of egg production and larval development are crucial. It should be noted that the one- and two-variable models explain a relatively low percentage of the variation in the observed series, the number of observations is small and some model coefficients have low levels of statistical significance. Detrending time series can also generate changes in the frequency of fluctuations and may have introduced some of the autoregressive variation in such short series.

The analyses highlight the differences between the biomass and abundance series, which refer to different geographical regions and year classes. The abundance data refer to the whole Atlantic sector, including the main recruitment regions of the southern Scotia Sea and around the Scotia Arc. Density-dependent effects in the abundance series may indicate that food availability or survival is affected by population size in the central recruitment regions of the southern Scotia Sea. The biomass series relate to a very local region on the northern edge of the distribution where the population is dependent on the influx of older age groups and density-independent factors have a stronger influence. The differences between the abundance and biomass series and analyses are further highlighted by the results that show that summer temperatures have a zero-lag positive influence on abundance but a negative impact on biomass. The negative influence of local temperatures on biomass is consistent with previous views that periods of low biomass occur when conditions are warm around South Georgia, which may reflect a reduced influence of cooler polar waters. The positive influence on abundance may reflect high abundance of young (0- or 1-year-old) age groups in the southern regions of the Scotia Sea when winter sea ice is less extensive across the region. The 2-year lag effects also show some differences in the analyses of biomass and abundance, which again are likely to reflect the different scales of the processes involved. The effects of sea ice fluctuations are positive and the same for biomass and abundance, in that more extensive sea ice leads to an increase in abundance and biomass 2 years later. This indicates an increase in abundance across the Scotia Sea and increased recruitment influx into the South Georgia region of animals that are 1 or 2 years old. However, warmer summer temperatures precede an increase in biomass 2 years later, whereas there is little evidence of an impact on abundance. The effect on biomass of a one-degree change in SST_−2_ is approximately five times as large as that for SST_0_, and in the opposite direction, indicating that local conditions 2 years earlier are crucial. This may in part reflect a rapid transition from warm to cold conditions in a cyclical system, which may lead to counter-intuitive relationships. However, the biomass data are local to South Georgia, where there are indications that warm periods are associated with reduced krill biomass and low recruitment (Murphy & Reid 2001).

Examining the factors influencing the rate of change in krill length between years gives similar results to those for biomass, indicating that physically driven fluctuations dominate the dynamics (see the electronic supplementary material). However, mean length shows consistent changes between years and is a significant predictive variable for change in length. Exploring length relationships in more detail, cross-correlation analyses of length against SST over the previous 36 months highlighted a strong negative relationship with SST 12–16 months earlier (figure 3*e*), and a strong positive relationship 23–28 months earlier, such that small krill dominated 2 years after very cold conditions (figure 3*f*). The smallest animals (mean length<43 mm) occurred in the diet of fur seals when SST in the previous summer was high, while the largest animals (mean length>48 mm) occurred when SST in the previous summer was coldest. As noted above, extreme lengths reflect periods of low biomass (figure 3*d*), and the influx of small animals (30–40 mm) into the population is also associated with interannual changes in biomass (figure 3*a*,*b*). We consider that this reflects a recruitment event following one or more years of little or no recruitment during a warm phase. Thus, good recruitment at South Georgia is easiest to detect at the beginning of the cold phase due to the absence of older age classes. Recruitment and a subsequent increase in biomass are therefore associated with cold periods (Trathan *et al*. 2003). These analyses are consistent with a view of a multi-age-class population with irregular fluctuations in recruitment (Murphy *et al*. 1998; Murphy & Reid 2001; Reid *et al*. 2002), which are linked with regional changes in oceanic conditions. The direct density-dependent effects reflect changes in age-class size resulting from fluctuations in recruitment, growth and mortality. Direct and indirect density-dependent effects of increased recruitment at low densities and reduced recruitment at high densities may also be occurring at a larger scale (Royama 1992).

Temperature can be important in determining krill development and growth rate both directly (through impact on physiological processes) and indirectly (through the availability of food; Atkinson *et al*. 2006). However, here we considered animals that ranged approximately from 30 to more than 55 mm and were between 1–2 and above 4 years old (Murphy & Reid 2001; Reid *et al*. 2002). Growth rate variation could be a contributory factor, but a consistent change in population size structure over a number of years indicates cohort effects.

To develop a more mechanistic understanding, and test this view of a multi-age-class population with recruitment linked to environmental changes, we used an age-structured model of the regional krill population to simulate the population changes. Based on the analyses of krill length in relation to SST (figure 3*e*,*f*), the model assumes the strength of a small subadult krill age class (30–40 mm, taken to be 2 years old in the simulation) to be a density-independent, nonlinear function of SST 1 or 2 years earlier. The model reproduces the major fluctuations in krill length observed in the diet of fur seals at South Georgia (figure 3*b*), but the outcomes are sensitive to the parametrization of the function relating recruitment to changes in the temperature of the previous season. The derived parametrizations tend to emphasize the nonlinear nature of the response to temperature, highlighting irregular recruitment events over a restricted range of SST. This analysis is consistent with the main conclusions of the regression analyses and supports the view that environment-driven recruitment fluctuation has dominated the population dynamics around South Georgia in recent years. The nonlinearity may in part be the result of density-dependent effects, but these are not included in the demographic model.

The analyses suggest a two-stage environmental impact on the dynamics of South Atlantic krill populations. First, a delayed effect of sea ice conditions 2 years earlier on abundance, which relates to the production, survival and development of the larval and juvenile krill (production recruitment). Second, there is a same year effect of spring and summer temperatures, which probably reflects a distribution and dispersal effect across the Scotia Sea and the degree of influence of cooler polar waters in northern regions around South Georgia (dispersal recruitment). The length of krill in the diet of fur seals at South Georgia is measured during March and can identify a dispersal recruitment event the season before the biomass changes (Reid *et al*. 1999; Murphy & Reid 2001). This is the probable explanation for why 1-year lag physical effects (SST fluctuations) are evident in the analyses of length and 2-year lag effects on changes in local biomass. Very warm periods of lowest recruitment tend, therefore, to precede the seasons of lowest biomass by 1 year and the next cold period and the associated recruitment and biomass increase by 2 years. The view that large-scale physical fluctuations affect recruitment across the Scotia Sea is also consistent with previous analyses, which indicate that changes in population structure and abundance occur in the same year across the Scotia Sea and Antarctic Peninsula regions (Murphy *et al*. 1998; Murphy & Reid 2001; Fach *et al*. 2002, 2006; Reid *et al*. 2002; Quetin & Ross 2003; Atkinson *et al*. 2004; Siegel 2005). The interpretation that fluctuations in krill recruitment around South Georgia are strongly physically driven may be particularly appropriate in this system for a number of reasons: (i) at present, krill abundance across the region is thought to be low compared with two to three decades ago (Atkinson *et al*. 2004), (ii) krill abundance across the northern Scotia Sea is thought to depend on dispersal which in turn is linked to the physical conditions, and (iii) the quasi-cyclic nature of the environmental variation is likely to result in marked interannual fluctuations in conditions for production and dispersal. The evidence that krill recruitment is also strongly linked to SST and sea ice conditions further strengthens the view that climate-related variation is an important control on krill abundance in the Scotia Sea region. However, this interpretation must be treated with some caution as the data series are short, the environmental data are cyclical and there are indications from the analyses of the large-scale abundance data that density-dependent processes may also be important. Such density-dependent population effects, including variation in mortality rate with population density, along with food web effects (such as competition of krill with salps or copepods) may contribute to the observed interannual fluctuations but are difficult to distinguish in such a short series (Royama 1992; Murphy *et al*. 2007). More complex models will be useful in exploring the detail of the mechanisms involved (Constable *et al*. 2003).

### (c) Krill and predator fluctuations

We also examined whether the changes in krill abundance and biomass affect the land-based predators. We found that fur seal pup production (pups surviving at the end of the breeding season; Forcada *et al*. 2005) is strongly associated with changes in krill size in the diet of lactating seals (figure 3*g*). Hence, the smallest krill occur in the diet at about the time of the lowest krill biomass, when pup production and survival are lowest. Weaning mass may be a better index of such short-term effects and analyses indicate that it is highly variable when krill biomass is low, but also show that the lowest weaning masses were recorded in years of extremely low krill biomass (figure 3*h*). This variation associated with low biomass estimates is not surprising given the known limitations of short-term (weeks) biomass surveys, which cannot capture the seasonal variability in krill biomass, but are considered to be useful in identifying years of very low biomass. Low pup production occurs at times when other krill-dependent predators (such as macaroni and gentoo penguins and black-browed albatross) also show low breeding performance (Croxall *et al*. 1988; Murphy *et al*. 1998). These analyses support the view that periods of reduced predator breeding performance are the result of low prey availability rather than direct local weather or oceanic effects (Croxall *et al*. 1988; Trathan *et al*. 2006). We conclude therefore that the observed correlation between breeding performance of a suite of krill predators and local SST (e.g. Trathan *et al*. 2006) is mediated by their prey. Krill recruitment failure takes a year to affect local krill biomass and availability to predators. This generates the observed 1 year delay between local SST variation and changes in predator breeding performance. Years of small krill size reflect a transition of low recruitment and declining krill biomass during warm years to a cold phase of good recruitment and a subsequent increase in krill biomass. We stress that owing to the nature of the functional response (e.g. pup production or weaning mass in relation to krill size or biomass: figure 3*g*,*h*) monitoring predator performance is much better suited to detecting years of low krill biomass, during the transition phases, than the subsequent periods of high biomass.

### (d) Climate interactions in ocean ecosystems

Together, the analyses of physical and biological data provide insight into some of the mechanisms involved in generating correlations between ENSO-related fluctuations in oceanic conditions across the equatorial Pacific and changes in higher predator biology in the Scotia Sea. The strong connections between sea ice and ENSO variability across the whole area result in correlations between ENSO variation and krill recruitment and also predator population dynamics (Murphy *et al*. 1998; Fraser & Hofmann 2003; Quetin & Ross 2003; see the electronic supplementary material for further discussion). As the sea ice variation is quasi-cyclic, we see related cycles in krill populations. Such quasi-cyclic environmental variation is a persistent feature in the Southern Ocean. Extended lifespans are an important strategy for organisms in such a variable environment where successful recruitment is intermittent (Murphy *et al*. 1998; Fraser & Hofmann 2003; Quetin & Ross 2003). It is therefore not surprising that krill are long lived (above 5 years). Such longevity allows the population to be maintained by 1 or 2 years of successful recruitment in each 4- or 5-year cycle (Murphy *et al*. 1998; Fraser & Hofmann 2003; Quetin & Ross 2003). These cycles in prey availability then generate periodic changes in predator breeding success, which generate long-term fluctuations in the predator population dynamics. The view of an influx of krill into the northern Scotia Sea ecosystem associated with current systems (Priddle *et al*. 1988; Murphy *et al*. 1998), and affected by climate-driven fluctuations in the ocean, is similar to the concept of a translation effect developed for *C. finmarchicus* in the North Atlantic (Pershing *et al*. 2004). Such large-scale ecosystem anomaly waves propagating physically in the oceans and biologically through food webs are likely to be a major component of ecosystem variation throughout the oceans.

During recent decades, there have been more El Niño than La Niña events, leading to a generally lower than average Southern Oscillation Index. This has prompted an active debate as to whether this change is climate driven and how it relates to other modes of climate variation such as the SAM that has also shown long-term change (Turner 2004). Since we have shown that variation across the southern Pacific affects Atlantic sector ecosystems, changes in the variation in the Pacific and across the Southern Ocean connected with climate change will be important in determining the long-term ecosystem dynamics (Stenseth *et al*. 2002; Quetin & Ross 2003; Ainley *et al*. 2005; Smetacek & Nicol 2005). However, direct effects of climate change in the Scotia Sea may also be important. The Antarctic Peninsula is one of the most rapidly warming regions on the planet and an area that is crucial in determining the dynamics of Scotia Sea krill populations (Murphy *et al*. 1998; Fach *et al*. 2002; Vaughan *et al*. 2003; Meredith & King 2005). Warming around the Antarctic Peninsula is associated with a reduced frequency of cold years of more extensive sea ice across the Scotia Sea (Murphy *et al*. 1995; Loeb *et al*. 1997; Fraser & Hofmann 2003). Meredith & King (2005) also showed that significant ocean surface warming (over 1°C) has occurred in regions to the west of the Antarctic Peninsula over the last 50 years. The significant decline in krill numbers recorded across the Scotia Sea over the last 30 years (Atkinson *et al*. 2004) is therefore likely to be the result of a reduced frequency of successful recruitment events associated with more frequent warm periods. However, long-term ecological effects resulting from harvesting of seals, whales and fishes over more than two centuries will also be occurring (Murphy 1995).

Reliable predictions of future change in complex ecological systems are difficult to make (Sutherland 2006). However, on the basis of the relationships revealed in this study, we would expect that, as a result of regional warming, conditions for dispersing krill across the Scotia Sea towards South Georgia will occur less frequently in the future. Predictions for the Scotia Sea region are not specifically available, but an increase of approximately 1–2°C in SST over the next century is possible and is a reasonable basis for prediction. Monte Carlo simulations of future change scenarios based on regression models that relate the rate of population growth with biomass or density in the previous 1 or 2 years and include a single environmental variable give similar results (table 1, M3 and M9; see electronic supplementary material for details). A rate of increase of 1°C over the next 100 years would result in more than 95% reduction in biomass in approximately 50–60 years (probability of local extinction *p*_e_=1.0). For abundance, the density-dependent effects are important and indicate that a more than 95% reduction in density could occur in less than 50 years (*p*_e_∼0.85). We also ran the models based on the CIs for the parameter values for increase rates of up to 2°C over the next 200 years. The outcomes indicate a high level of uncertainty and suggest that local extinction may occur any time throughout next 2 centuries, but the population could be maintained through density-dependent effects. The models do not include the direct effects of temperature on individual growth rates, analyses of which indicate that an increase in SST of 1°C across the northern Scotia Sea would significantly reduce growth rates (Atkinson *et al*. 2006), potentially further reducing population viability.

The simulations highlight the uncertainty in such predictions and indicate a high degree of sensitivity of the system to local warming, with potential catastrophic effects on krill and their dependent predators across the region. It should be noted that SST increases of this order do not require large extrapolation of mean values beyond the range of temperatures currently observed (at 34.5°W, 54.5°S, the mean SST (September to March)=1.60°C, max.=3.65, min.=−0.48, s.d.=1.24). In reality, the responses of krill populations and their predators to oceanic temperature change are likely to be highly nonlinear. Indeed, the analyses show that potential density-dependent effects cannot be ignored. For such oceanic climate-driven local extinction not to occur requires nonlinearities in krill dynamics at low densities, such that low levels of recruitment do not continue during extended periods of high temperature. One possibility is that density-dependent population effects further south, in the main region of krill production, may become more important at low densities of krill. However, on the basis of our current understanding of the dynamics of krill populations and model simulations, we suggest that further regional warming could produce a more than 95% decline in krill biomass and abundance across the Scotia Sea during the next 100 years (cf. Atkinson *et al*. 2004). Reducing the uncertainty associated with these predictions by developing better mechanistic models of krill life cycles coupled with physical circulation models is an urgent requirement.

## Figures and Tables

**Table 1:** Multiple linear regression models for krill population growth rate based on the South Georgia biomass and the South Atlantic abundance (residual) series. (SST, sea surface temperature anomaly during spring (end of September); SSTS, sea surface temperature anomaly during summer (end of December); Ice, maximum ice extent at 45°W during winter; IceC, ice concentration anomaly at 45°W during winter=(concentration over winter in year *t*−mean concentration over all winters); *B*_*t*_, krill biomass (g m^−2^) in year *t*; *X*_*t*_, residual value of the detrended series of krill density in year *t*; *N*_*t*_, krill density (N m^−2^) in year *t*. n.s., not significant (5% level); ^*^, significant one-tailed test; ^**^, significant two-tailed test. Models were estimated for all possible combinations of variables (number of data points=10). The five models with the lowest Akaike Information Criteria (AIC_c_) values are reported.)

	model equation								
			
*model* (*M*)	*krill biomass ln*(*B*_*t+*1_/*B*_*t*_)							*R*^*2*^ (*%*)	*ΔAIC*_c_
1	0.04^n.s.^	+4.74^**^	SST_−2_					76	4.57
2	−0.07^n.s.^	+4.23^**^	SST_−2_	−0.79^**^	SSTS_0_			92	0.00 (19.84)
3	3.10^**^	−1.00^**^	ln(*B*_0_)	−3.35^**^	SST_0_			87	3.64
4	1.98^*^	−0.60^*^	ln(*B*_0_)	+3.69^**^	SST_−2_			86	4.52
5	18.44^n.s.^	+5.59^**^	SST_−2_	−0.67^**^	SSTS_0_	+0.32^n.s.^	Ice_−2_	94	4.45
	*numbers* (*X*_*t*+1_−*X*_*t*_)								
6	1.75^*^	−0.82^**^	ln(*N*_0_)					42	4.42
7	2.94^**^	−0.75^**^	ln(*N*_0_)	−0.54^*^	ln(*N*_−1_)			61	4.90
8	1.44^*^	−0.62^*^	ln(*N*_−1_)	+1.18^**^	SSTS_0_			58	5.43
9	33.44^**^	−0.74^**^	ln(*N*_0_)	−0.93^**^	ln(*N*_−1_)	+0.51^**^	Ice_*−*2_	87	0.00 (26.61)
10	2.54^**^	−1.06^**^	ln(*N*_−1_)	+2.08^**^	SST_−1_	+20.91^**^		79	4.64

## Supplementary Material

Climate impacts in ocean ecosystems Extended methods and analyses
