# Supplementary material for: Climatically driven fluctuations in Southern Ocean ecosystems
Source: Proc Biol Sci. 2007 Oct 17;274(1629):3057–67. doi: 10.1098/rspb.2007.1180 (PMC2211519; doi:10.1098/rspb.2007.1180)
Supplement: Supplementary file 1 [file rspb20071180s06.pdf]

Electronic supplementary material

Climatically driven fluctuations in Southern Ocean ecosystems

Eugene J. Murphy[*1], Philip N. Trathan[1], Jon L. Watkins[1], Keith Reid[1], Michael P. Meredith[1], Jaume Forcada[1], Sally E. Thorpe[1], Nadine Johnston[1] & Peter Rothery[2]

[1]*British Antarctic Survey, Natural Environment Research Council, High Cross, Madingley Road, Cambridge, Cambridgeshire, CB3 0ET, U. K.*

[2]*Centre for Ecology and Hydrology, Natural Environment Research Council, Monks Wood, Abbots Ripton, Huntingdon, Cambridgeshire, PE28 2LS. UK.*

Supplementary Methods and Analyses

Physical data. The timing of the variables used in the analyses are illustrated schematically in supplementary table 1.

Biological data. Demographic data on Antarctic fur seals (termed fur seals) from Bird Island, South Georgia include estimates of the number of pups produced each year during the breeding season and the mass at which they were weaned. Pup production is considered to be the best index of interannual changes in predator performance while weaning mass gives an index of performance during the early part of the season (Forcada *et al.* 2005). Weekly data on the mean length of krill (in mm) consumed by fur seals at South Georgia were collected since 1991. The mean length of krill in the diet during the last 3 wks in March is used as an index of krill population changes (Reid *et al.* 1999). Acoustic data providing short-term (~1 wk) estimates of local (within < 100 km distance of Bird Island, 80 x 80 km region) krill biomass were collected irregularly between 1981 and 1994 and yearly between November and January since 1995.

**Year $_{-2}$**	**Year $_{-1}$**	**Year $_{0}$**	**Year $_{+1}$**
	B_{-1}	B_0	B_{+1}
	N_{-1}	N_0	N_{+1}
	L_{-1}	L_0	L_{+1}
SST_{-2}	SST_{-1}	SST_0	
$SSTS_{-2}$	$SSTS_{-1}$	$SSTS_0$	
Ice_{-2}	Ice_{-1}	Ice_0	
$IceC_{-2}$	$IceC_{-1}$	$IceC_0$	

Supplementary Table 1. Schematic illustration of timing of the variables used in the regression analyses. Definitions: *SST* = Sea surface temperature anomaly during spring (end of September); *SSTS* = Sea surface temperature anomaly during summer (end of December); *Ice* = Maximum sea ice extent on 45°W during winter; *IceC* = Ice concentration anomaly on 45 ^{o}W during winter = (concentration over winter *t* - mean concentration over all winters). B_t = krill biomass (g m^{-2}) in year *t*; N_t = krill density (N m^{-2}) in year *t*; L_t = krill length (mm) in year *t*.

Time series analyses. To account for the autocorrelation structure for cross-correlation analyses shown here, the monthly SST and sea ice data were analysed with a seasonal decomposition based on a semiparametric regression with loess smoothing (Cleveland *et al.* 1990; Forcada *et al.* 2005). The seasonal component was removed from each monthly series and the new sub-series was smoothed to find long-term trends through several iterations. The residuals of the seasonal and trend fits were also removed, leaving a smoothed trend filtered for residual noise. The filtered series showed the succession of anomalies over time and was used in subsequent cross-correlation analyses (supplementary figure 1). The ice-edge position at 45.5^{o}W (15% concentration) was used as an index of sea ice cover in this area (supplementary figure 2).

Pacific and South Atlantic variation in SST

Further to the analyses given in the main text (see paper figure 2) the detailed analyses of the interannual changes in SST at South Georgia and their relationship with the south-east Pacific sector and equatorial region SST variation are presented in

supplementary table 2 and are plotted in supplementary figure 2. Smoothing the data to remove monthly variation indicated that 49% of the variation in SST anomalies at South Georgia (SG) was explained by a model including both the SST anomaly data from the Amundsen-Bellingshausen Sea (AB) region 12 months previously and the Nino-3 series 6 months previously (Based on smoothed 6-month moving average series; $SG_0 = 0.020 + 0.382\ AB_{-12} - 0.160\ \text{Nino-3}_{-6}$; $p < 0.0001$). Scotia Sea ice extent shows marked inter- and intra-annual variation, which is closely correlated with changes in SST (supplementary figure 3). As noted in the main text the Scotia Sea sea ice variation leads the South Georgia SST fluctuations by < 6 months.

	R^2 (%)	Model (n = 255)	ΔAIC_c	n_{adj}	R^2_S
1	21	$-0.025^{NS} + 0.582^{**}\ BS_{-10}$	148.03	90	21
2	15	$0.163^{**} - 0.674^{**}\ E4_{-4}$	323.43	76	19
3	17	$0.053^{NS} - 1.043^{**}\ E3_{-4}$	519.78	77	16
4	28	$0.000^{NS} + 0.287^{**}\ BS_{-10} - 0.111^{**}\ E3_{-4}$	5.49	83	26
5	28	$0.021^{NS} + 0.297^{**}\ BS_{-10} - 0.161^{**}\ E4_{-4}$	5.78	82	29
6	23	$-0.010^{NS} + 0.359^{**}\ BS_{-10} + 0.034^{**}\ SA_0$	22.66	94	24
7	30	$-0.005^{NS} + 0.284^{**}\ BS_{-10} - 0.111^{**}\ E3_{-4} + 0.033^{**}\ SA_0$	0.00 (189.54)	86	29
8	30	$0.013^{NS} + 0.278^{**}\ BS_{-10} - 0.095^{**}\ E4_{-4} - 0.067^{**}\ E3_{-4}$	2.73	81	29
9	30	$0.016^{NS} + 0.298^{**}\ BS_{-10} - 0.152^{**}\ E4_{-4} + 0.026^{**}\ SA_0$	3.30	84	30

Supplementary Table 2. Multiple linear regression models of fluctuations in SST anomalies at South Georgia. Initial cross correlation analyses identified maximum correlation lags. The 3 regressions for the same number of variables with the lowest values of AIC_c are shown. Variables are SST anomaly for the Bellingshausen Sea (BS) region for 10 months earlier; Nino-4 series 4 months earlier, Nino-3 series 4 months earlier and the SAM monthly series with no lag. ΔAIC_c values are given for comparable models and scaled from the lowest value of AIC_c. n_{adj} = adjusted sample number allowing for series autocorrelation; Spearman Rank Correlation (R_S). All regressions are significant (< 1% probability level) as are correlation coefficients (including autocorrelation effects).

Population dynamics of krill in the Scotia Sea

Regression modelling. Linear regression models were estimated for all possible combinations of variables and the 5 models with the lowest Akaike Information Criteria (AIC_c) were reported in the paper table 1. Here in each model case, for the same

number of independent variables, the 3 (or less where fewer were significant) models with lowest AIC_c values are reported (supplementary table 3). To allow for non-normality we calculated Spearman's Rank Difference Correlation coefficients (r_s) as well as Pearson's Product-Moment Correlation coefficients (r). The number of observations was also adjusted for autocorrelation using the equation: $n_e = n/(1 + 2\ r_{a1}\ r_{b1} + 2\ r_{a2}\ r_{b2})$, where r_{a1} = series a correlation lag 1, r_{a2} = series a correlation lag 2, r_{b1} = series b correlation lag 1, r_{b2} = series b correlation lag 2 (Ottersen & Loeng 2000; Ottersen & Stenseth 2001).

	Model equation		
Model number (M)	*krill biomass* ($\ln[B_{t+1}/B_t]$)	R^2 (%)	ΔAIC_c
1	$0.04^{NS} + 4.74^{**}\ SST_{-2}$	76 (*,-)	4.57
2	$3.75^{**} - 1.18^{**}\ \ln(B_0)$	53 (-,-)	10.76
3	$-0.25^{NS} - 3.95^{**}\ SST_0$	50 (-,-)	11.28
4	$-0.07^{NS} + 4.23^{**}\ SST_{-2} - 0.79^{**}\ SSTS_0$	92 (*,*)	0.00 (19.84)
5	$3.10^{**} - 1.00^{**}\ \ln(B_0) - 3.35^{**}\ SST_0$	87 (*,*)	3.64
6	$1.98^{*} - 0.60^{*}\ \ln(B_0) + 3.69^{**}\ SST_{-2}$	86 (*,*)	4.52
7	$18.44^{NS} + 5.59^{**}\ SST_{-2} - 0.67^{**}\ SSTS_0 + 0.32^{NS}\ Ice_{-2}$	94 (*,*)	4.45
8	$9.76^{NS} + 3.79^{**}\ SST_{-2} - 0.82^{**}\ SSTS_0 + 0.17^{NS}\ Ice_0$	93 (*,*)	5.13
9	$-0.03^{NS} + 4.70^{**}\ SST_{-2} - 0.74^{**}\ SSTS_0 + 4.08^{NS}\ IceC_{-2}$	93 (*,*)	5.23
	numbers ($X_{t+1} - X_t$)		
10	$1.75^{*} - 0.82^{**}\ \ln(N_0)$	42 (-,-)	4.42
11	$-0.10^{NS} + 1.22^{*}\ SSTS_0$	34 (-,-)	5.69
12	$2.94^{**} - 0.75^{**}\ \ln(N_0) - 0.54^{*}\ \ln(N_{-1})$	61 (-,-)	4.90
13	$1.44^{*} - 0.62^{*}\ \ln(N_{-1}) + 1.18^{**}\ SSTS_0$	58 (*,-)	5.43
14	$2.34^{**} - 0.99^{**}\ \ln(N_{-1}) + 18.00^{*}\ IceC_{-2}$	55 (-,-)	6.13
15	$33.44^{**} - 0.74^{**}\ \ln(N_0) - 0.93^{**}\ \ln(N_{-1}) + 0.51^{**}\ Ice_{-2}$	87 (*,*)	0.00 (26.61)
16	$2.54^{**} - 1.06^{**}\ \ln(N_{-1}) + 2.08^{**}\ SST_{-1} + 20.91^{**}\ IceC_{-2}$	79 (*,*)	4.64
17	$3.40^{**} - 0.61^{*}\ \ln(N_0) - 0.90^{**}\ \ln(N_{-1}) - 2.12^{NS}\ SST_{-2}$	73 (*,*)	7.11
	length (ΔLt)		
18	$0.48^{NS} + 6.78^{**}\ SSTS_{-2}$	73 (*,*)	6.68
19	$52.36^{**} - 1.15^{**}\ L_0$	56 (*,*)	12.09
20	$-0.37^{NS} - 5.10^{**}\ SSTS_{-1}$	43 (-,-)	14.93
21	$46.39^{**} - 1.02^{**}\ L_0 - 4.37^{**}\ SSTS_{-1}$	87 (*,*)	2.44
22	$-90.69^{**} + 6.23^{**}\ SSTS_{-2} - 1.57^{**}\ Ice_{-2}$	86 (*,*)	3.68
23	$0.18^{NS} - 2.37^{NS}\ SSTS_{-1} + 5.57^{**}\ SSTS_{-2}$	80 (*,*)	7.30
24	$47.79^{**} - 1.06^{**}\ L_0 - 5.63^{**}\ SSTS_{-1} - 32.50^{**}\ IceC_{-1}$	94 (*,*)	0.00 (51.95)
25	$-197.35^{**} - 11.37^{**}\ SST_{-2} + 6.75^{**}\ SSTS_{-2} - 3.40^{**}\ Ice_{-2}$	92 (*,*)	2.89
26	$-13.24^{NS} - 1.00^{**}\ L_0 - 3.62^{**}\ SSTS_{-1} - 1.01^{*}\ Ice_{-2}$	92 (*,*)	3.05

Supplementary Table 3. Multiple linear regression models for krill population growth rates based on the South Georgia krill biomass (BAS data) and South Atlantic abundance, and for change in mean length of krill in the diet of lactating Antarctic fur seals (March). Definitions: *SST* = Sea surface temperature anomaly during spring (end of September); *SSTS* = Sea surface temperature anomaly during summer (end of December); *Ice* = Maximum ice extent at 45°W during winter; *IceC* = Ice concentration anomaly at 45 °W during winter = (concentration over winter in year *t* - mean concentration over all winters). B_t = krill biomass (g m^{-2}) in year *t*; X_t = residual value of the detrended series of krill density in year *t;* N_t = krill density (N m^{-2}) in year *t*; L_t = krill length (mm) in year *t*. ΔL_t = change in krill length (mm) from year *t* to *t+1*. NS = not significant (5% level), * = significant 1-tail test, ** = significant 2-tail test. Models were

estimated for all possible combinations of variables. For each set of models the change in AIC_c (ΔAIC_c) is reported relative to the model with the lowest value (shown in brackets). The R^2 values (%) for the models are shown with an indication of the significance level in brackets of the derived values of the Pearson's Product Moment and Spearman Rank Difference correlation coefficients based on the model predictions compared to the observations with sample size adjusted to allow for autocorrelation in both series (* = significant 5%, - = not significant).

Krill population dynamics model. The krill population at South Georgia is not self-sustaining, but is maintained by inputs of post-larval krill from further south. Recruitment at South Georgia therefore refers to the influx into the population of older (1 to 2 year old) individuals. A demographic krill model was used to model changes in the mean length of krill in the South Georgia population (Murphy & Reid 2001). Changes in the number of animals in each year class was assumed to be determined by a constant mortality rate between years ($N_{t+1} = N_t\, e^{-M}$ where N_t is the number of animals in year t and M is the instantaneous rate of natural mortality). The relationships shown in figures 3e and 3f (main paper) relate krill length to SST in earlier years and we assume reflect changes in recruitment strength associated with temperature. We derived 2 different non-linear functional relationships of recruitment strength with SST over the previous 2 seasons. For comparison, SST was taken as the anomaly at the end of December in both cases. The relationships were used to model changes in recruitment strength and to predict mean length of krill in March to allow comparison with fur-seal diet analyses (paper figure 3b).

For the relationship with SST in the previous season, the relative abundance (RI_t) of the juvenile cohort that has survived the first winter (here termed year class 1+) was estimated as a function of the SST anomaly (T_t) at the end of December of the previous season and is expressed as:

$$RI_t = 1 - \left(1 + e^{\beta(T_d + T_t)}\right)^{-1} \qquad 1$$

The second function relates recruitment of the larval cohort (0+ age group) to the SST anomaly (T_t) at the end of December 2 years before the observed South Georgia recruitment and is expressed as:

$$RI_t = \left(1 + e^{\beta(T_d + T_t)}\right)^{-1} \quad 2$$

Where β is a constant and T_d is the SST anomaly that results in a relative abundance index of 0.5. Changes in the population were then calculated in both cases based on all year classes 2 yrs or older. The functions then produce either a 1 or 2 -year lag between the environmental effect of SST on the size of the recruiting age class and its effect on the population as the 2 yr age class.

Changes in the mean size of each krill year class were modelled as a seasonal von Bertalanffy function. A Normal distribution (mean = 0, sd = 2.5) was used to derive a cohort size distribution (2 mm length resolution) for each year (Murphy & Reid 2001). The complete length frequency was then derived as the sum across all year classes. The mean length was derived from the combined length distribution (Reid *et al.* 1999). Multiple simulations were produced in a Monte Carlo analysis to estimate the best fit von Bertalantffy parameters k (growth coefficient) and L_α (maximum krill length in mm) and the mortality rate with an analysis of least-squares. Other parameter values used in the von Bertalanffy function were $t_0 = 0.2$, $t_s = -0.5$ and $c = 0.9598$ (Reid *et al.* 2002). The model fitting was insensitive to the values of M and L_α over a realistic range of parameters (0.6 to 2 per annum and 58 to 67 mm respectively), but was sensitive to the value of k. Estimates of this parameter are highly variable. For comparison values of 1.25 and 61 mm for M and L_α were used in both models. Values of k between approximately 0.45 and 0.55 gave a similar goodness of fit so k was taken as 0.5 in both cases. The model runs were sensitive to the parameterisation of the recruitment function and only a restricted set of parameter values gave a recruitment series that could reproduce the observed changes in krill length. Values of $\beta = 5$ and $T_d = -0.05$ °C were used for the 1-year lag model (*Equation 1*). Values of $\beta = 5$ and $T_d = 0.5$ °C were used for the 2-year lag model (*Equation 2*). These parameterisations tend to enhance the effects of warm (*Equation 1*) or cold (*Equation 2*) years.

Climate interactions in ocean ecosystems.

Climate impacts in ocean ecosystems. Further to the discussion given in the paper, here we summarise the links between climate related fluctuations in the Pacific and the ecosystem impacts in the South Atlantic. Large-scale climate-related processes in the equatorial Pacific generate anomalies in SST across the South Pacific sector of the Southern Ocean that propagate in the Antarctic Circumpolar Current. This physical variation can be further modified by direct short term (< 6 month) ENSO and SAM related atmospheric effects. This variation impacts krill population processes in the southern Scotia Sea. This generates biological fluctuations that in turn propagate across the Scotia Sea and up to higher trophic levels in the regional ecosystem. The nature of the relationship is such that positive SST anomalies in the ENSO regions occur around 2 - 3 years prior to positive anomalies in the southwest Atlantic; this variability then generates a biological response-wave in the regional ecosystem (see supplementary figure 4). When the Scotia Sea is in a cold phase strong recruitment occurs which generates a peak in krill abundance and dispersal of older year classes 1 - 2 years later, and a maximum krill biomass in the north after a further 1 - 2 years. The physical signal is sufficiently strong in the southeast Pacific 1 year before it enters the Scotia Sea that it will be a useful basis for prediction. We can therefore predict that warm conditions (positive anomalies) in the southeast Pacific to the west of the Peninsula will lead the Scotia Sea region by 1 year, and hence pre-empt periods of very low krill biomass and poor predator breeding performance across the northern Scotia Sea by 2 years.

Forecasting the effects of climate change. To consider the potential effects of regional warming on krill population dynamics we firstly undertook Monte Carlo simulations of future change scenarios. The simulations were based on the multiple regression models shown in the main table 1. For these long-term projections we used the lowest AIC_c values models which included a zero-lag population term and a single environmental variable. These relate the rate of population growth for biomass with B_t and SST (in the spring) (paper table 1, M3), and for density include terms N_t and N_{t-1} and ice conditions with a 2-year lag (paper table 1, M9). We assume that the models represent the major factors controlling krill density and biomass in the Scotia Sea. Applying these models in this way does not take account of the effects of other long-

term physical and biological interaction effects, such as changing circulation patterns, food-availability, competition or predation. The models were used to estimate the change in the population growth rate (based on density and biomass) and hence population size over the next 100 years. The SST anomaly was represented as a Normal variate with mean 0 and standard deviation of 0.26, estimated from the satellite derived series from 1982 to 2006 for 34.5^{o}W and 54.5^{o}S. The sea-ice extent (maximum northward 15% ice edge in winter) was represented as a Normal variate with based on the mean position at 58.15 ^{o}S and with a standard deviation of 1.14, estimated from the satellite derived series on 45°W. The initial krill density was taken to be 15 individuals m^{-2} and biomass as 25 g m^{-2}. In each case the model was run 1000 times. This was then repeated, but with a trend (ΔC ^{o}C y^{-1}) included in the SST series. For the biomass model this gives:

$$R_t = \alpha + \beta_1 \ln(B_t) + \beta_2 (\Delta C\, t + \varepsilon_t)$$

Where R_t is the population growth rate ($\ln[B_{t+1}/B_t]$) at time t, B_t is the biomass and α, β_1 & β_2 are the model parameters (paper table 1, M3) and ε_t is the Normal variate representing the SST variation. Model M9 included a delay term (N_{t-1}) and a lag effect of sea-ice. For each set of runs we estimated the probability of the population size (density or biomass) being reduced and remaining at a level less than 5% of the start value during the simulation. The results were not sensitive to the threshold level selected. The probability of decline of the krill population was estimated as the proportion of the 1000 runs in which the decline occurred. For these runs we also estimated the mean time at which the decline below 5% occurred. We note that in this case the reduction refers to local decline of krill across the Scotia Sea/South Atlantic sector of the Southern Ocean and not to the whole krill population. However, as this region currently contains ~50% of the circumpolar population of krill such a change would have profound consequences.

Further to the points made in the main text, a simplistic extrapolation of the declining trend in the abundance series, if it continues, would suggest an absence of krill from the Scotia Sea in about 25 to 30 years. Developing more detailed predictions of biological responses to global climate change are limited by uncertainty in both physical system

changes and the biological processes involved. Climate models generally predict warming and sea ice reductions in the Southern Ocean over the next 100 years, but the magnitude of these predicted changes is highly variable between models (Clarke *et al.* 2007). Current coupled ocean-atmosphere models do not predict well the rapid regional atmospheric warming that has occurred around the west Antarctic Peninsula over the last 50 years.

An increase in frequency of years of low production, through a change in the periodicity and/or an increase in mean SST, would tend to increase the potential for locally significant reduction in krill abundance. The demographic age-structured model generates a very similar effect. The form of the recruitment functional relationship (we consider the 2-year-lag model, see above) indicates that an increase of 1°C would lead to consistently low recruitment and reduce abundance by > 95% in < 100 years. Derived relationships of predator performance and SST also suggest that changes of only ~1°C would have a significant negative impact on predator breeding performance (Forcada *et al.* 2005; Trathan *et al.* 2006).

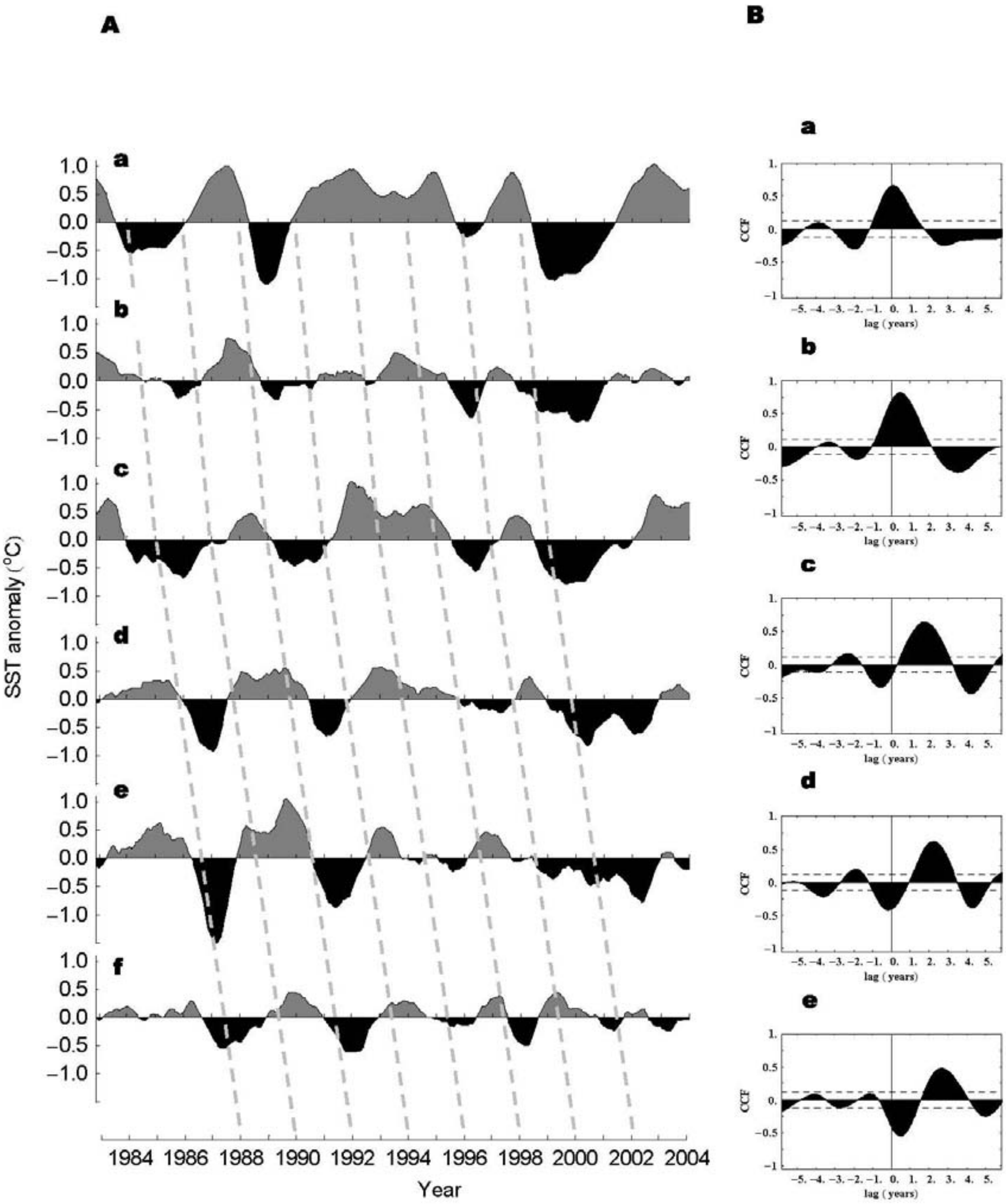

Supplementary Figure 1. Spatial and temporal relationships of SST anomalies. A) Spatial and temporal changes in warm and cold SST anomalies in the a) ENSO Nino-4 region and at 45° longitudinal points along 60 ^{o}S, b) 179.5 ^{o}W, c) 134.5 ^{o}W, d) 89.5 ^{o}W, e) 69.5 ^{o}W and f) south east of South Georgia (34.5 ^{o}W, 54.5 ^{o}S) in the area where the SACCF penetrates into the South Georgia (SG) region(Trathan & Murphy 2002). Diagonal dashed lines (grey) show a delay of 1 year from the ENSO region to the central south Pacific sector and a 2 year delay from the central south Pacific sector to the South Georgia region. B) Cross correlation functions (CCF) of the ENSO Nino-4 region series with SST anomalies at 45° points along 60 ^{o}S, a) 179.5 ^{o}W, b) 134.5 ^{o}W, c) 89.5 ^{o}W, d) 69.5 ^{o}W, and e) south east of South Georgia. Nino-4 leads for positive lag.

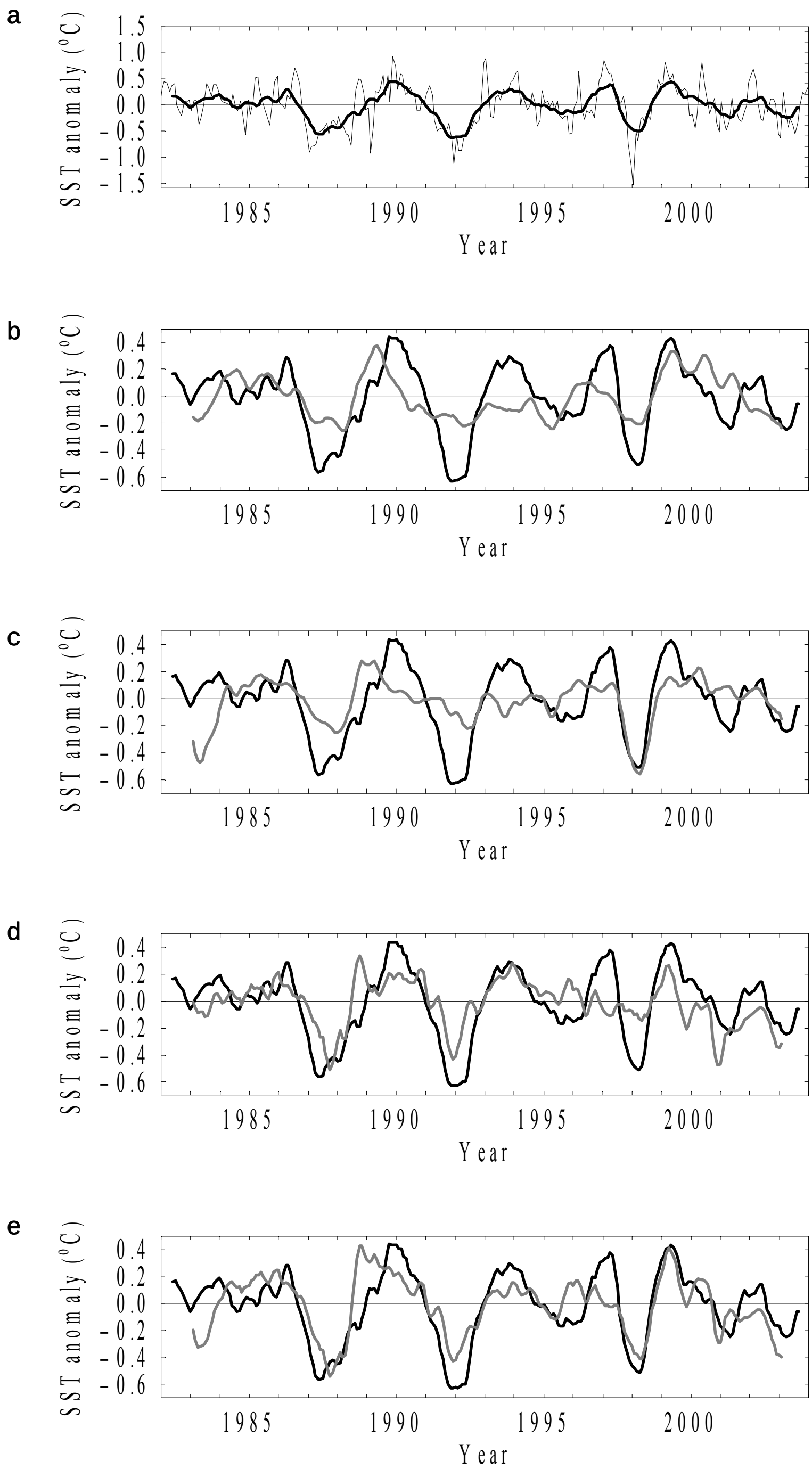

Supplementary figure 2. Predicted sea surface temperature (SST) anomaly series at South Georgia derived by multiple-regression analysis. a) South Georgia SST anomaly series (thin line) with the 12 month moving average series (black). Smoothed South Georgia series (black line) with predicted series (grey line) based on the regression relationships with the: b) Nino-4 series lagged by 4 months, R^2 = 15%, c) Nino-3 series lagged by 4 months, R^2 = 17%, d) Bellingshausen Sea series (89.5°W, 60°S) lagged by 10 months, R^2 = 21%, e) all 3 series, lagged Bellingshausen Sea series (89.5°W, 60°S), Nino-4 and Nino-3 region series , R^2 = 30%. For all regressions $n = 255$ and $p < 0.001$. A 4 month moving average was applied to predicted series to remove high frequency monthly variability for plotting.

a

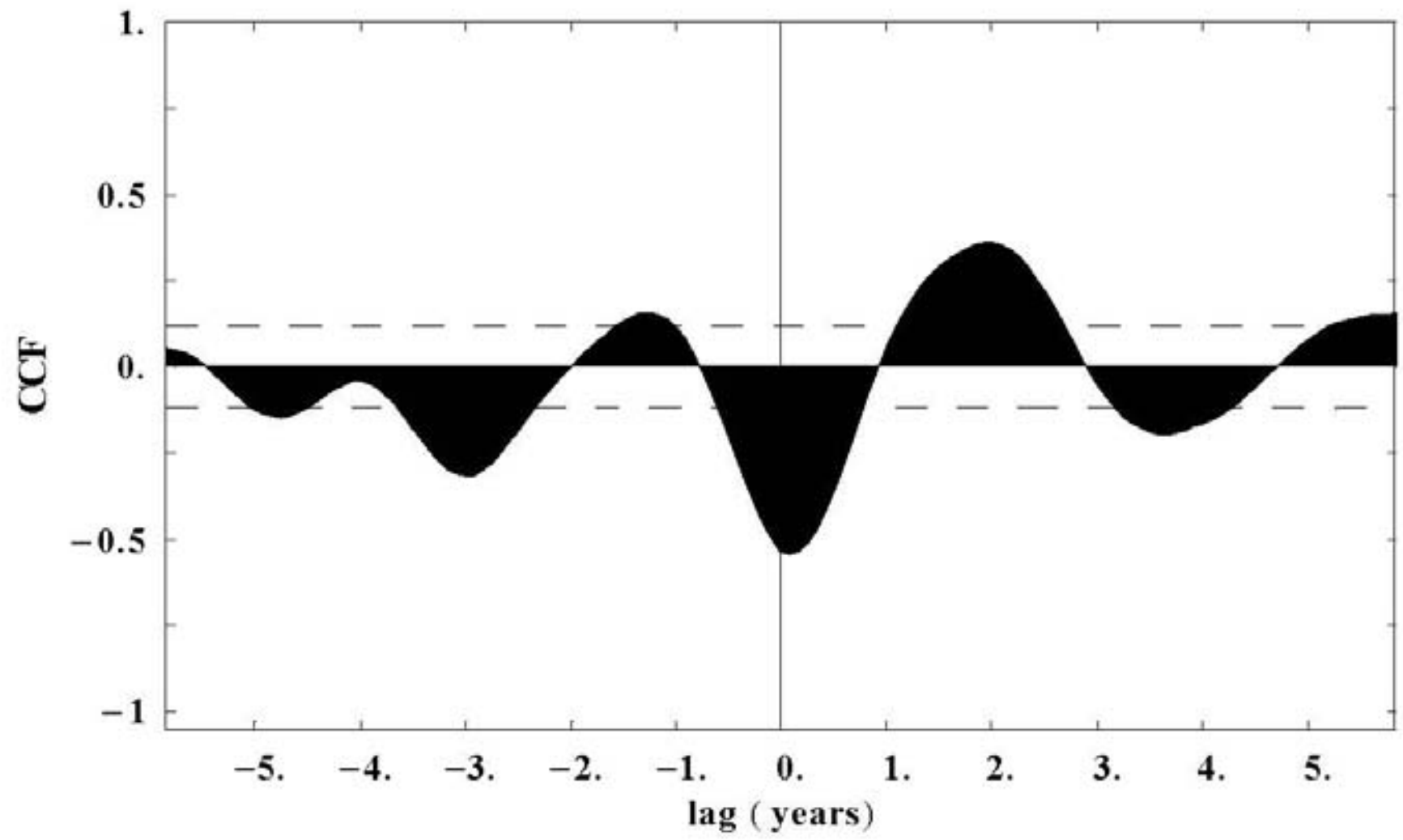

b

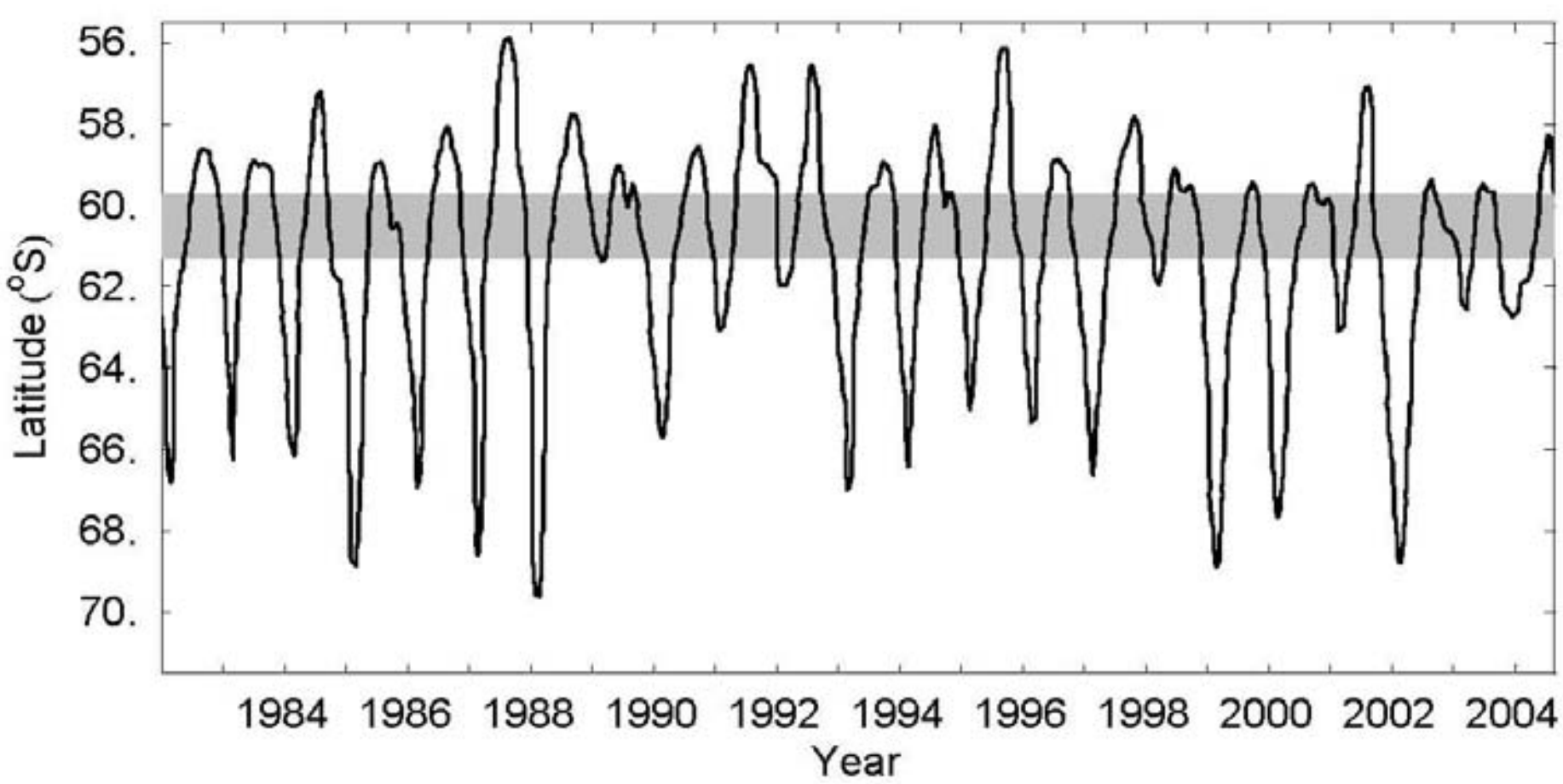

Supplementary Figure 3.

a) Cross correlation functions (CCF) for the areal sea ice concentration anomaly on 45.5°W (positive anomaly = sea ice concentration greater than average) and the South Georgia SST anomaly series. b) Northern limit of sea ice extent (15% concentration) on 45.5°W between 1982 and 2004. The grey band shows the approximate position of the South Orkney Island shelf (< 500 m deep).

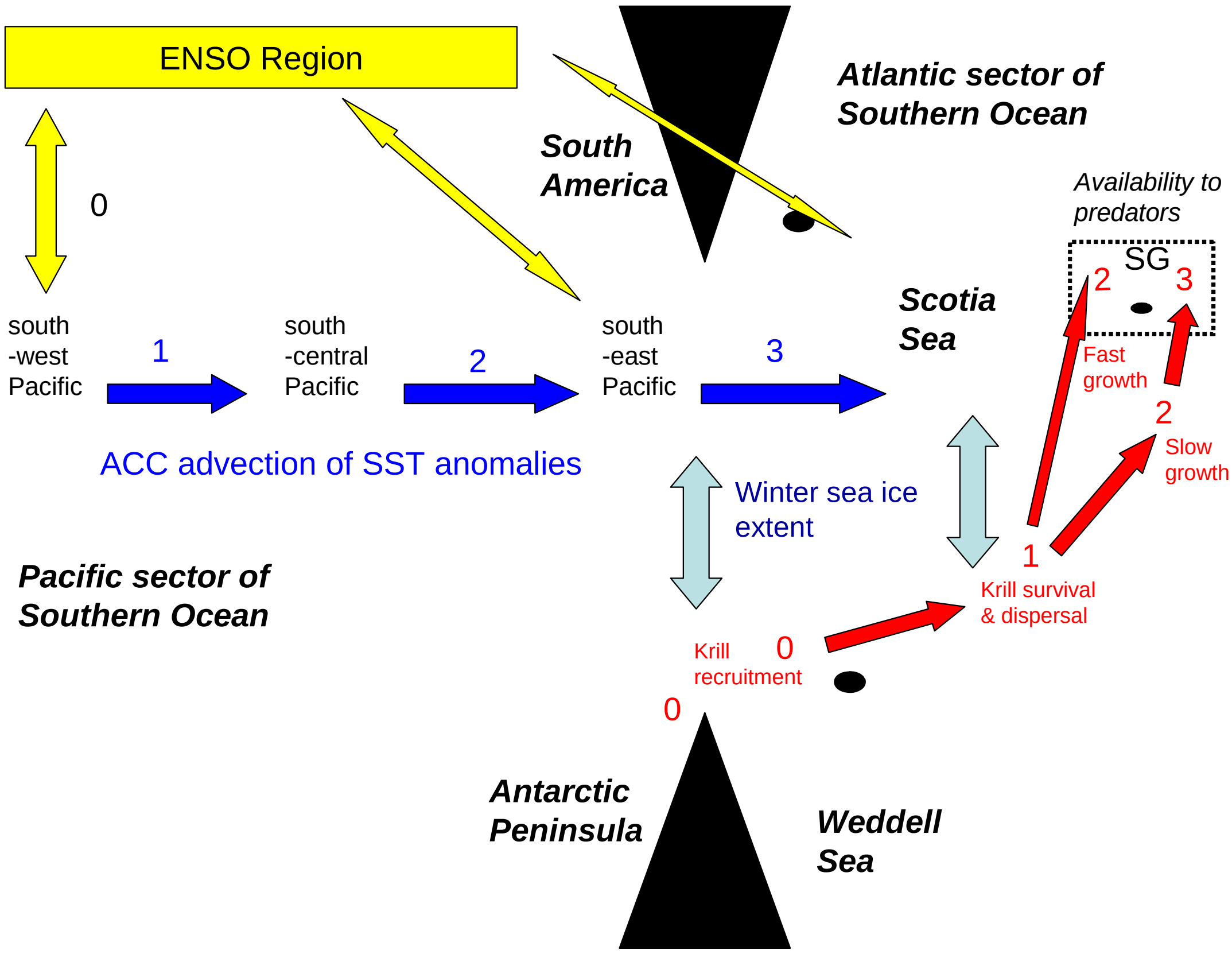

Supplementary Figure 4. Schematic of physical and biological links generating variations in the age structure and distribution of Antarctic krill (*Euphausia superba*) populations across the Atlantic sector of the Southern Ocean (SO) ecosystem. Anomalies in Sea Surface Temperature (SST) initiated by the El Niño Southern Oscillation (ENSO) propagate from the Pacific sector of the SO into the Atlantic sector of the SO in association with the Antarctic Circumpolar Current (ACC) over a period of 2 to 3 years, with other atmospheric forcing acting on the anomalies as they propagate. Regional changes in SST are linked to fluctuations in winter sea ice in the southern Scotia Sea and around the Antarctic Peninsula. The distribution of sea ice affects the recruitment, survival and dispersal of Antarctic krill. Physically driven variation in krill population dynamics and abundance, in turn affects the breeding success of seabird and marine mammal predators that depend on krill as food. There is no lag between SST variations in the ENSO region and the south west Pacific sector of the SO (yellow arrow). Once generated, anomalies in the southwest Pacific take around 2-3 years to propagate to the southwest Atlantic, with significant further atmospheric interaction occurring as they progress (especially in the southeast Pacific, yellow arrows). Colder SST periods in the Atlantic sector associated with more extensive winter sea ice (light blue arrows) enhance Antarctic krill recruitment in this region (i.e. more 0-age class individuals) and generates higher winter krill-survival rates over the following 1 or 2 winters. More northward extent of sea ice also enhances the dispersal of these krill further across the Scotia Sea towards South Georgia (SG) (red arrows) where they become the dominant size class in the diet of Antarctic fur seals (*Arctocephalus gazella*) in the following season. Depending on the growth conditions experienced after spawning, these krill may be 2 to 3 years old by the time they reach South Georgia. Numbers in red refer to the age class of krill in the various regions.

Supplementary References

Clarke, A., Murphy, E. J., Meredith, M. P., King, J. C., Peck, L. S., Barnes, D. K. A. & Smith, R. C. 2007 Climate change and the marine ecosystem of the western Antarctic Peninsula. *Philosophical Transactions of the Royal Society B-Biological Sciences* **362**, 149-166.

Cleveland, R. B., Cleveland, W. S., McRae, J. E. & Terpenning, I. 1990 STL: A Seasonal-Trend Decomposition Procedure Based on Loess. *Journal of Official Statistics* **6**, 3-73.

Forcada, J., Trathan, P. N., Reid, K. & Murphy, E. J. 2005 The effects of global climate variability in pup production of Antarctic fur seals. *Ecology* **86**, 2408 – 2417.

Murphy, E. J. & Reid, K. 2001 Modelling Southern Ocean krill population dynamics: biological processes generating fluctuations in the South Georgia ecosystem. *Marine Ecology-Progress Series* **217**, 175-189.

Ottersen, G. & Loeng, H. 2000 Covariability in early growth and year-class strength of Barents Sea cod, haddock, and herring: the environmental link. *Ices Journal of Marine Science* **57**, 339-348.

Ottersen, G. & Stenseth, N. C. 2001 Atlantic climate governs oceanographic and ecological variability in the Barents Sea. *Limnology and Oceanography* **46**, 1774-1780.

Reid, K., Murphy, E. J., Loeb, V. & Hewitt, R. P. 2002 Krill population dynamics in the Scotia Sea: variability in growth and mortality within a single population. *Journal of Marine Systems* **36**, 1-10.

Reid, K., Watkins, J. L., Croxall, J. P. & Murphy, E. J. 1999 Krill population dynamics at South Georgia 1991-1997, based on data from predators and nets. *Marine Ecology-Progress Series* **177**, 103-114.

Trathan, P. N. & Murphy, E. J. 2002 Sea surface temperature anomalies near South Georgia: Relationships with the Pacific El Nino regions. *Journal of Geophysical Research-Oceans* **108**, art. no.-8075.

Trathan, P. N., Murphy, E. J., Forcada, J., Croxall, J. P., Reid, K. & Thorpe, S. E. 2006 Physical forcing in the southwest Atlantic: ecosystem control. In *Top Predators in Marine Ecosystems: Their Role in Monitoring and Management.*, vol. 12 (ed. I. L. Boyd, S. Wanless & C. J. Camphuysen), pp. 28-45: Cambridge University Press.
